# Supplementary material for: Co-Inoculation of Plant-Growth-Promoting Bacteria Modulates Physiological and Biochemical Responses of Perennial Ryegrass to Water Deficit
Source: Plants (Basel). 2022 Sep 28;11(19):2543. doi: 10.3390/plants11192543 (PMC9570635; doi:10.3390/plants11192543)
Supplement: Supplementary file 1 [file plants-11-02543-s001.zip › plants-1931858-supplementary.pdf]

Supplementary material

Co-inoculation of plant growth-promoting bacteria modulate physiological and biochemical responses of perennial ryegrass to water deficit

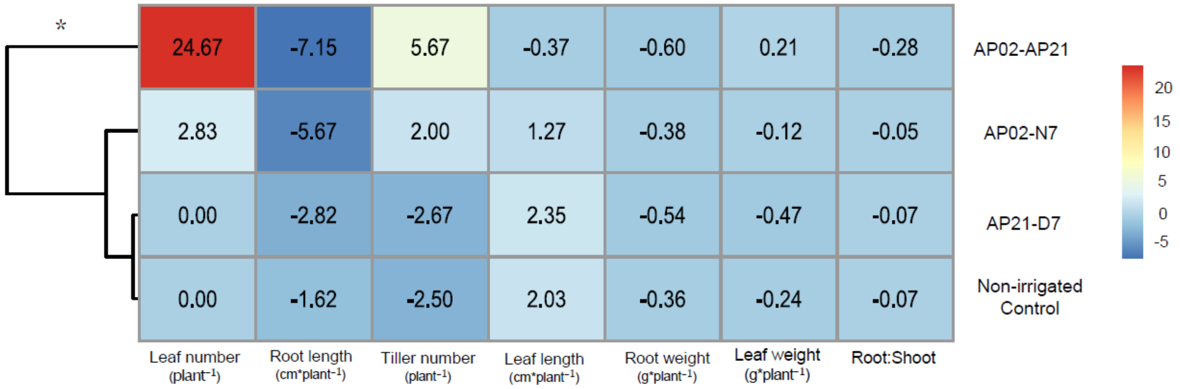

Figure S1. Heatmap and multiple comparison test for the morphological parameters of perennial ryegrass. The variables were analyzed via gDGC multiple comparison of means with significance level α = 0.05 and ward linkage (asterisk shows a significant difference) and validated with a Hotelling test with the Bonferroni correction ($p > 0.05$).
